# Gene expression of vascular endothelial growth factor A and hypoxic adaptation in Tibetan pig

**DOI:** 10.1186/s40104-016-0082-z

**Published:** 2016-04-02

**Authors:** Bo Zhang, Yangzong Qiangba, Peng Shang, Yunfeng Lu, Yuzeng Yang, Zhixiu Wang, Hao Zhang

**Affiliations:** National Engineering Laboratory for Animal Breeding, China Agricultural University, Beijing, 100193 People’s Republic of China; College of Agriculture and Animal Husbandry, Tibet University, Linzhi, 860000 People’s Republic of China; School of life science & technology, Nanyang normal University, Nanyang, 473061 Henan Province People’s Republic of China; Hebei Provincial Husbandry and Veterinary Research Institute, Baoding, Hebei 071001 People’s Republic of China

**Keywords:** Gene expression, Hypoxic adaptation, Tibetan pig, *VEGFA* gene

## Abstract

**Background:**

Vascular endothelial growth factor A (*VEGFA*) can induce endothelial cell proliferation, promote cell migration, and inhibit apoptosis. These processes play key roles in physiological blood vessel formation and pathological angiogenesis.

**Methods:**

In this study, we examined *VEGFA* gene expression in the heart, liver, and kidney of Tibetan pigs (TP), Yorkshire pigs that migrated to high altitudes (YH), and Yorkshire pigs that lived at low altitudes (YL). We used PCR and Sanger sequencing to screen for single nucleotide polymorphisms (SNPs) in 5ʹ-flanking DNA and exons of the VEGFA gene. Quantitative real-time PCR and western blots were used to measure expression levels and PCR products were sequenced.

**Results:**

Results showed that the *VEGFA* mRNA and protein expression in heart, liver and kidney of TP was higher than that in YH and YL. In addition, the mRNA sequence of the pig *VEGFA* gene was conserved among pig breeds, and only five SNPs were found in the 5ʹ-flanking region of the *VEGFA* gene, the allele frequency distributions of the 5 SNPs were not significantly different between the TP, Yorkshire (YL), and Diannan small-ear (DN) pig populations.

**Conclusion:**

In conclusion, the Tibetan pig showed high levels of *VEGFA* gene expression in several hypoxic tissues, which suggests that the *VEGFA* gene may play a major functional role in hypoxic adaptation.

## Background

*VEGFA* (also known as *VEGF*) is a major growth factor for endothelial cells. It promotes vascular permeability and angiogenesis by stimulating proliferation, migration, and survival of endothelial cells, as well as inhibiting apoptosis [1–3]. *VEGFA* ligand binding to *VEGFRs* upregulates expression of endothelial nitric oxide synthase (*eNOS*) and increases prostacyclin production in endothelial cells [4], and is strongly expressed in antiproliferative lesions from patients with severe primary idiopathic and secondary forms of pulmonary hypertension [5, 6]. In pig, the *VEGFA* gene maps to chromosome seven, comprises seven exons, and has one transcript.

Tibetan pig (TP) is indigenous to China and live primarily in semi-agricultural and semi-pastoral areas (average elevation: 2500–4300 m) in the Qinghai-Tibet Plateau of southwest China. The TP have adapted to harsh conditions such as hypoxia [7–9], which makes this species a good model for investigating molecular mechanisms of hypoxic adaptation.

Hypoxia is a potent inducer of *VEGFA* through regulation of hypoxia-inducible factors (*HIF*s). However, the function and mechanism for hypoxic adaptation in TP remain unclear. The objective of the present study was to detect expression of the *VEGFA* gene in different tissues including the heart, liver, and kidney from three groups of pigs living at different altitudes. This study should help elucidate the function of the *VEGFA* gene in hypoxic adaptation of Tibetan pig.

## Methods

The experimental processes were approved by the animal welfare committee of the State Key Laboratory for Agro-biotechnology of China Agricultural University (Approval number XK257), and pig farming at Linzhi of Tibet is permitted and the field study does not involve endangered or protected species.

### Experimental materials

Experiments were performed using pigs from three different populations: Tibetan pig from highlands (Linzhi, 3,000 m) (TP), Yorkshire pig that migrated to high altitude (Linzhi, 3,000 m) (YH), and Yorkshire pig raised at lowland (Beijing, 100 m) (YL). Animals in the YH group were descended from a population of Yorkshire pigs that migrated from lowland to highland approximately 3 yr ago. Ten castrated boars from each population were slaughtered when they were 6 mo of age. Tissue samples were collected from the liver, heart, and kidney and were immediately frozen in liquid nitrogen. Samples were then stored at -80 °C.

Ear tissue samples were collected from three pig populations: YL from the Beijing Shunxinlong Farm (*n* = 30), TP from Linzhi, Tibet of China (*n* = 60), and Diannan small-ear (DN) from Xishuang Banna, Yunnan of China (*n* = 40). The samples were immediately frozen and stored at -20 °C.

### DNA, RNA, and protein extraction and cDNA preparation

Genomic DNA was isolated from ear tissue as previously described [10], dissolved in TE solution, and stored at -20 °C.

Total RNA was extracted from the heart, liver, and kidney with TRIZOL^®^ Reagent (Invitrogen, San Diego, CA, USA), checked for concentration and purity using a NanoDrop 2000 Biophotometer (Thermo Fisher Scientific Inc., West Palm Beach, FL, USA), and separated by electrophoresis in a 1 % agarose gel to verify integrity. After treatment with DNase I, 2 μg of RNA in a 20 μL reaction volume was reversely transcribed into cDNA using a SuperRT cDNA Kit (CWBIO Ltd., Beijing, China).

Total protein was isolated from the heart, liver, and kidney using SDS Lysis Buffer (P0013B, Beyotime Ltd., China). Protein content was measured with the enhanced BCA protein assay kit (P0010, Beyotime, Ltd., China).

### SNP screening and genotyping

Primers for identification of SNPs in the *VEGFA* gene (NM_214084) were based on DNA sequence obtained using the UCSC BLAT Search Genome tool (http://genome.ucsc.edu/). We used the amplified pig mRNA sequence and Primer Premier 5.0 software to design primers that amplified the coding regions (exons 1 to 7) and 5ʹ-flanking sequences of the gene. The targeted regions, primer sequences, and amplicon sizes are shown in Table 1. PCR products amplified from 10 pigs in each group were pooled and sequenced to identify SNPs. Chromas Pro and DNAMAN6.0 were used to analyze the sequencing data. Genotypes of SNPs found by pooling sequencing were determined with individual PCR and sequencing.

**Table 1:** Target region, sequence, and amplicon size of the primers used for SNP identification

Primer	Target region	Forward primer sequence (5ʹ to 3ʹ)	Reverse primer sequence (5ʹ to 3ʹ)	Amplicon size, bp
5ʹ- FR1	−1902/−2693	AGTGACTGGCTCCTGTTCTC	CCTGGGTAGAAGTATTTGGC	791
5ʹ- FR2	−2193/−1902	CGTTCCTTAGTGCTGGTGAG	AAAGTGAGGTTATGTGCGGC	843
5ʹ- FR3	−1546/−631	GTGTGTCTGGGTGTGTGTGG	TCCCTCTCGTTTCTTGCTTGC	915
5ʹ- FR4	−654/+53	GGGCAAGCAAGAAACGAGA	AGGTAGAGCAGCAAGGCAA	707
*VEGFA*-P1	Exon1	GAGGAGGAAGAAGAGAAGGAAG	CATGTACGAGGATAGAGGGGAA	472
*VEGFA*-P2	Exon2	CCATTCTTCCCTCTTTGTTTTGTC	TTTGTTTTCCCAGTCTGTGCTCA	367
*VEGFA*-P3	Exon3	GGCCGGCCCCCTCTACAG	AACGGGCTTTTTAAACTCTCCACA	630
*VEGFA*-P4	Exon4-5	CCTGGTCTGTGGAGAGTTTA	AGTGGGTAGAGAAAGAGAAA	872
*VEGFA*-P5	Exon6	CTGCCGCTCTCTCTTGTCTTCTGC	AGCCACGCCTGCCACCTG	564
*VEGFA*-P6	Exon7	CGTAGGGACTCTTCTTTGGT	CTCGGCTTGTCACATCTGC	313

### Quantitative analysis of *VEGFA* mRNA expression

To avoid genomic DNA contamination, we used Primer Premier 5.0 software to design *VEGFA* gene (NM_214084) primers that amplified products spanning an intron. The primers were 5ʹ-GAGGAGTTCAACATCGCCAT-3ʹ and 5ʹ-GAGGAGTTCAACATCGCCA-3ʹ. We used the housekeeping gene glyceraldehyde-3-phosphate dehydrogenase (*GAPDH*, NM_001206359) as the internal standard and the primers were 5ʹ-GGTCACCAGGGCTGCTTTTA-3ʹ and 5ʹ-CCTTGACTGTGCCGTGGAAT-3ʹ. Quantitative real-time PCR (qRT-PCR) was conducted on the Bio-Rad CFX96 System (Bio-Rad, USA). Each reaction mixture contained 10.0 μL 2× SYBR Green qPCR SuperMix (Transgen, Beijing, China), 1.0 μL cDNA, 0.5 μL of each primer (10.0 nmol/μL), and ddH_2_O water to adjust the volume to 20.0 μL. The real-time PCR program started with denaturation at 95 °C for 20 s. This was followed by 40 cycles of denaturation at 95 °C for 5 s and annealing/elongation at 60 °C for 15 s, during which fluorescence was measured. Next, a melting curve was constructed by increasing the temperature from 65 °C to 95 °C in sequential steps of 0.5 °C for 5 s, during which fluorescence was measured. The real-time PCR efficiency of each pair of primers was calculated using 5 points in a 5-fold dilution series of cDNA, which was used to construct a standard curve. A cDNA pool of all samples was used as a calibration and three replications of each sample were performed. Gene expression levels were calculated using the 2^-△△Ct^ method (△△Ct = △Ct _target gene_ - △Ct _housekeeping gene_) as previously described [11].

### Western blotting

Approximately 30 mg of each tissue used in quantitative real-time PCR was homogenized in lysis buffer (10 mmol/L NaH_2_PO_4_, 1 mmol/L EDTA, 10 mmol/L β-mercaptoethanol, 0.25 % Triton X-100, and 0.02 % NaN_3_, adjusted to pH 6.8). Tissues were homogenized using a Mixer Mill MM400 (Retsch, Germany) for 5 min and then centrifuged at 10,000 × *g* for 10 min at 4 °C. Protein concentrations were determined using a Protein Assay Kit (Bio-Rad). Proteins (40 μg) were separated by sodium dodecyl sulfate polyacrylamide gel electrophoresis (SDS-PAGE) using a 5 % stacking gel and a 10 % separating gel. Following electrophoresis, proteins were transferred to Immobilon-P Transfer Membranes (IPVH00010) for 2 h at 300 mA using a Bio-Rad Criterion Blotter. Membranes were blocked overnight in blocking buffer (P0023B, Beyotime Ltd., China) and then incubated with primary mouse monoclonal *GAPDH* (1:1,000 dilution, AG019, Beyotime Ltd., China), and *VEGFA* (1:500 dilution, LS-C2929, LifeSpan BioSciences, Seattle, WA) antibodies diluted in primary antibody dilution buffer (P0023A, Beyotime Ltd., China) at 4 °C for 2 h. After 3 washes with PBST(phosphate buffer saline containing 0.1 % Tween 20), membranes were incubated with secondary HRP-labeled goat anti-mouse IgG (H + L) (1:1,000 dilution, A0216, Beyotime Ltd., China) antibody diluted in secondary antibody dilution buffer (P0023D, Beyotime Ltd., China) for 1 h. After the membranes were washed 3 times in Tris-buffered saline with Tween for 30 min, immune complexes were visualized using an eECL Western Blot Kit (CW0049A, CWBIO Ltd., China) according to the manufacturer’s instructions. To determine expression ratios of *VEGFA* and *GAPDH*, western blots were analyzed using Image J 1.44 software (NIH, USA).

### Cell culture

Cell culture reagents were obtained from GIBCO (Life Technologies, Lofer, Austria). PIEC (KG302, KeyGEN BioTECH, China) were cultured according to the manufacturer’s instructions. Experiments were performed using two incubators. For normoxia treatments, one incubator (Thermo Fisher Scientific Inc., West Palm Beach, FL, USA) was set at 37 °C and 5 % CO_2_; the incubator oxygen sensor indicated approximately 21 % O_2_. Cells were cultured under normoxic conditions for 2, 4, 8, 12, 24, or 36 h. For hypoxia treatments, an incubator (3 gas incubator, Changsha Hua Xi Electronics Technetronic Co., Ltd., China) was set at 37 °C, 5 % CO_2_, and 94 % N_2_; the oxygen sensor indicated approximately 1 % O_2_. Cells were cultured under hypoxic conditions for 2, 4, 8, 12, 24, or 36 h. Cells were collected after the indicated durations in culture and total RNA extraction, cDNA synthesis and qTR-PCR were performed as described above.

### Statistical analyses

Expression levels were analyzed by one-way ANOVA using SAS9.1 Software (SAS Inst. Inc., Cary, NC). Graphs were prepared using SigmaPlot 10.0 (Systat Software, San Jose, CA) and data are presented as mean ± standard error. Significant and extreme differences were set at *P* < 0.05 (*) and *P* < 0.01 (**), respectively.

## Results

### SNPs and genotype frequencies

**Fig. 1:**
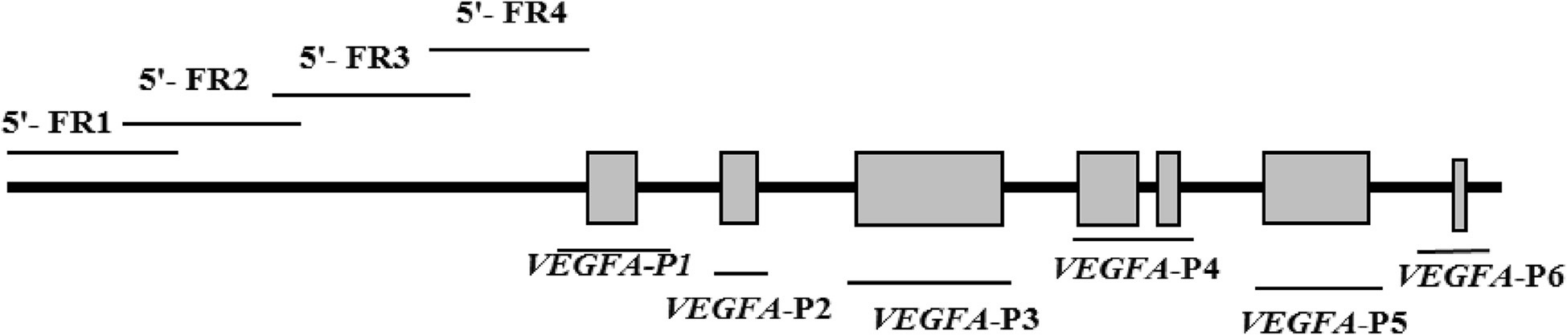
Structure of the pig *VEGFA* gene and the positions of primers used for SNP identification. The thick black lines represent flanking regions and introns; the grey blocks represent exons of the *VEGFA* gene; the thin black lines represent positions of amplicons. Pig total DNA was used as PCR templates for the 5ʹ-FR1, 5ʹ-FR2, 5ʹ-FR3, 5ʹ-FR4, *VEGFA*-P1, *VEGFA*-P2, *VEGFA*-P3, *VEGFA*-P4, and *VEGFA*-P5 primers

**Fig. 2:**
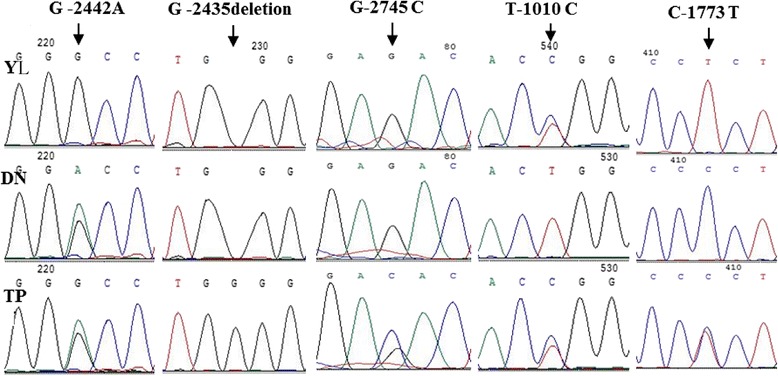
Sequencing chromatograms of 5 SNPs: G-2442A, G-2435 deletion, G-2745C, T -1010C, and C-1773 T. Chromatogram of the PCR product amplified using the 5ʹ-FR1 primer set (Table 1) shows the 3 identified SNPs G-2442A, G-2435 deletion, and G-2745 C. The 5ʹ-FR2 primer set (Table 1) shows SNP C-1773 T. The 5ʹ-FR3 primer set (Table 1) shows SNP T-1010C. YL = Yorkshire pig (*n* = 30), DN = Diannan small-ear pig (*n* = 40), TP = Tibetan pig (*n* = 60)

The structure of the pig *VEGFA* gene and the positions of the primers used for SNP identification are shown in Fig. 1. Using the primers listed in Table 1, the PCR amplicons covered 2,693 bp of the 5ʹ-flanking and full-coding regions (all 7 exons). No SNPs were detected in the coding region of the *VEGFA* gene among the TP, YL, and DN populations. Sanger sequencing revealed 5 SNPs at upstream 2,435, 2,442, 2,745, 1,010, and 1,773 bp from the initiation codon of the *VEGFA* gene that were named G-2745C, G-2442A, G-2435deletion, T-1010C and C-1773 T respectively (Fig. 2).

Individual sequencing analysis indicated genotype and allele frequencies of the 5 SNPs in the 3 pig populations (Table 2). No significant differences in genotypes distributions at loci G-2745C, G-2442A, and G-2435deletion were seen comparing TP with YL or DN (*P* > 0.05). Although the TP had a different genotype distribution in T-1010C with the DN, the difference between TP and YL was not significant (*P* > 0.05). At locus C-1773 T, there were significant differences in genotype frequency comparing TP with YL or DN; however, the allele C frequency of TP was between YL and DN.

**Table 2:** Gene and genotype frequency of the 5 SNPs in different pig breeds

Loci	Breed	Genotype (number/percentage)				Allele	
		GG	GC	CC	*P* value* (Fisher’s exact test)	G	C
G-2745C	YL	10/1	0/0	0/0	0.237	1	0
	DN	10/1	0/0	0/0	0.237	1	0
	TP	8/0.800	2/0.200	0/0		0.900	0.100
		GG	GA	AA		G	A
G-2442A	YL	10/1	0/0	0/0	0.500	1	0
	DN	9/0.900	1/0.1	0/0	0.763	0.950	0.050
	TP	9/0.900	1/0.1	0/0		0.950	0.050
		GG	G-deletion	Deletion		G	Deletion
G-2435 deletion	YL	8/0.889	1/0.111	0/0	0.474	0.940	0.060
	DN	7/0.700	0/0	3/0.300	0.105	0.700	0.300
	TP	10/1	0/0	0/0		1	0
		TT	TC	CC		T	C
T-1010C	YL	35/0.875	5/0.125	0/0	0.342	0.938	0.062
	DN	40/1	0/0	0/0	0.010	1	0
	TP	16/0.8	4/0.2	0/0		0.900	0.100
		CC	CT	TT		C	T
C-1773 T	YL	1/0.034	7/0.233	22/0.733	0.000	0.150	0.850
	DN	36/1	0/0	0/0	0.001	1	0
	TP	14//0.700	4/0.200	2/0.100		0.800	0.200

Note: *P value was significance of the exact test for genotype frequency distribution compared with TP. YL = Yorkshire pig (*n* = 30), DN = Diannan small-ear pig (*n* = 40), TP = Tibetan pig (*n* = 60)

### *VEGFA* mRNA expression

**Fig. 3:**
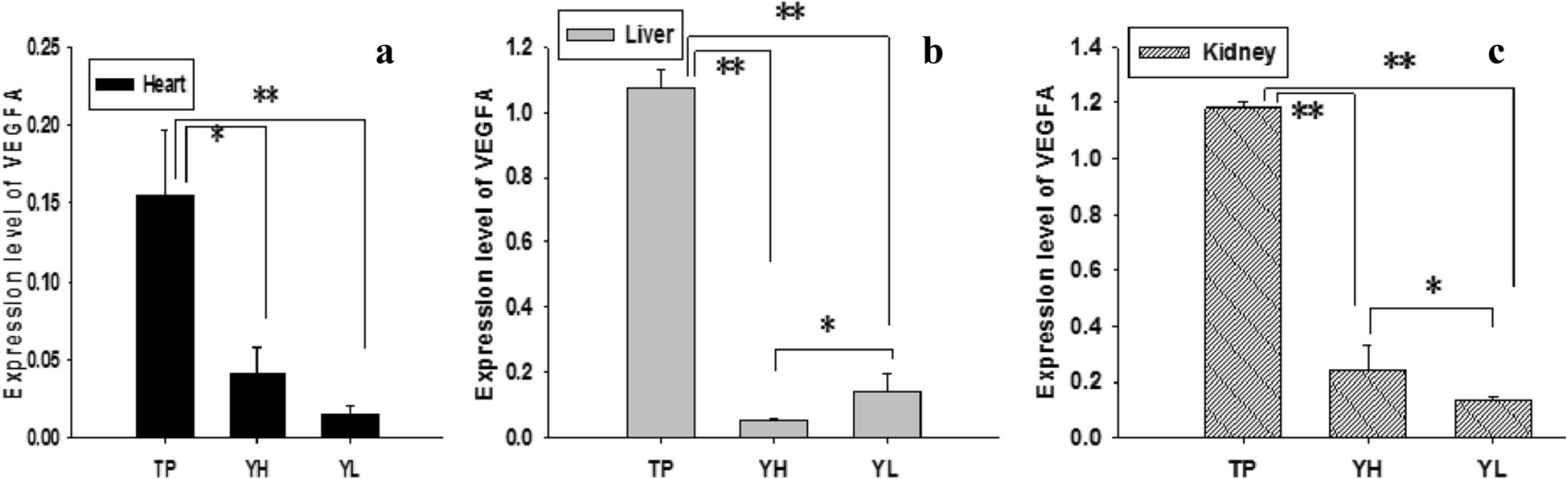
Expression of *VEGFA* mRNA in the heart (**a**), liver (**b**), and kidney (**c**). Each bar represents mean ± S.E. * Significant difference (*P* < 0.05), ** Extreme significant difference (*P* < 0.01). TP = Tibetan pig (*n* = 10); YH = Yorkshire pig raised at high-altitude (*n* = 10); YL = Yorkshire pig raised at lowland (*n* = 10)

PCR efficiencies of *VEGFA* and *GAPDH* genes were within 95 to 105 % that was satisfied for qRT-PCR. Expression of *VEGFA* mRNA is shown in Fig. 3. We found that expression of *VEGFA* mRNA was relatively high in the liver and kidney, but low in the heart. Moreover, under hypoxic conditions, expression of *VEGFA* mRNA in all three tissues was significantly higher in TP than in YH and YL (*P* < 0.01). Following migration of Yorkshire pigs from lowland to highland, expression of *VEGFA* mRNA increased in the kidney (*P* < 0.05), but trended downward in the liver.

### *VEGFA* protein expression

**Fig. 4:**
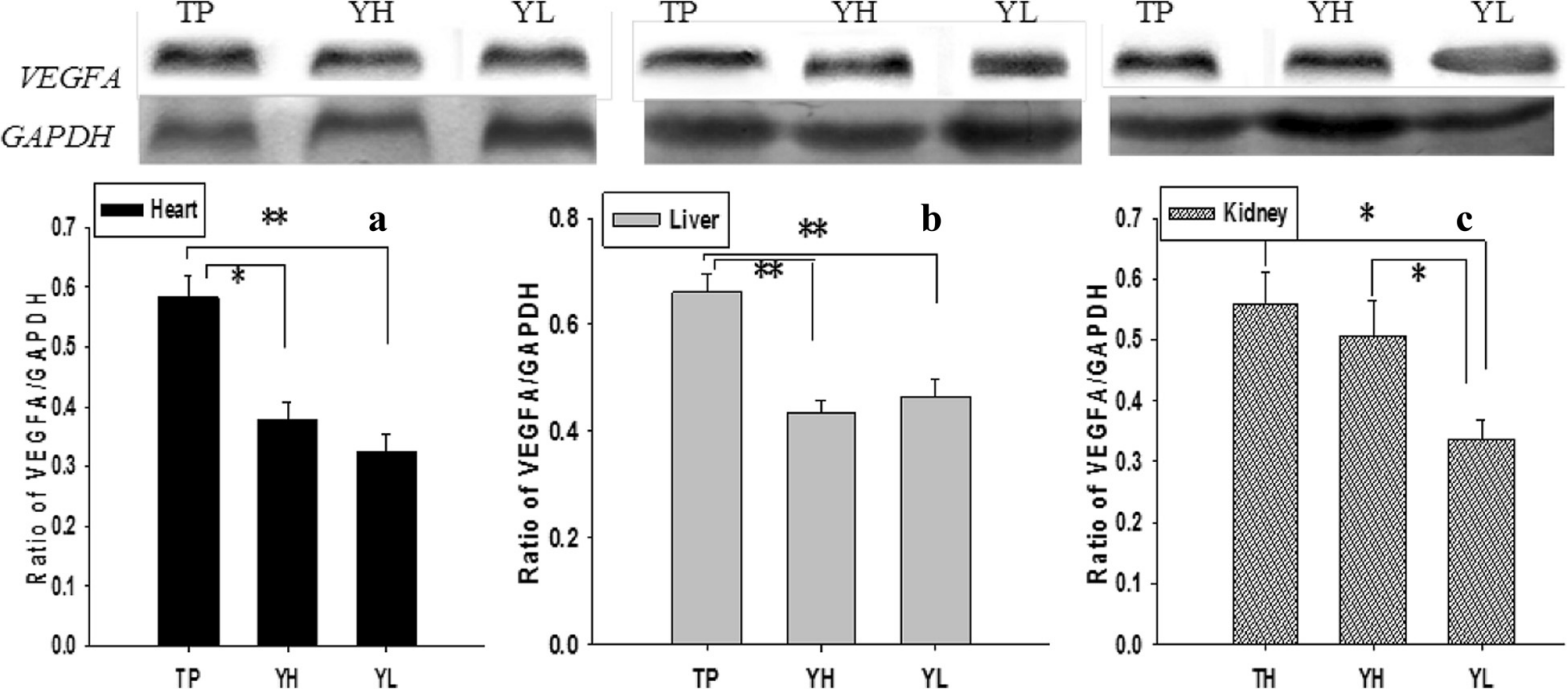
Expression of *VEGFA* proteins in the heart (**a**), liver (**b**), and kidney (**c**). Each bar represents mean ± S.E. * Significant difference (*P* < 0.05), ** Extreme significant difference (*P* < 0.01). TP = Tibetan pig (*n* = 10); YH = Yorkshire pig raised at high-altitude (*n* = 10); YL = Yorkshire pig raised at lowland (*n* = 10)

Results western blot showed that the *VEGFA* protein expression had same difference trends in heart, liver and kidney with mRNA expression between the three groups (Fig. 4). The protein expression was significantly higher in heart and liver of TP than that of YH and YL (*P <* 0.05). While in kidney tissue, the TP had higher *VEGFA* protein expression than YL (*P <* 0.05) and YH, although the difference between TP and YH was not significant (*P >* 0.05).

### *VEGFA* gene expression in PIEC cells

**Fig. 5:**
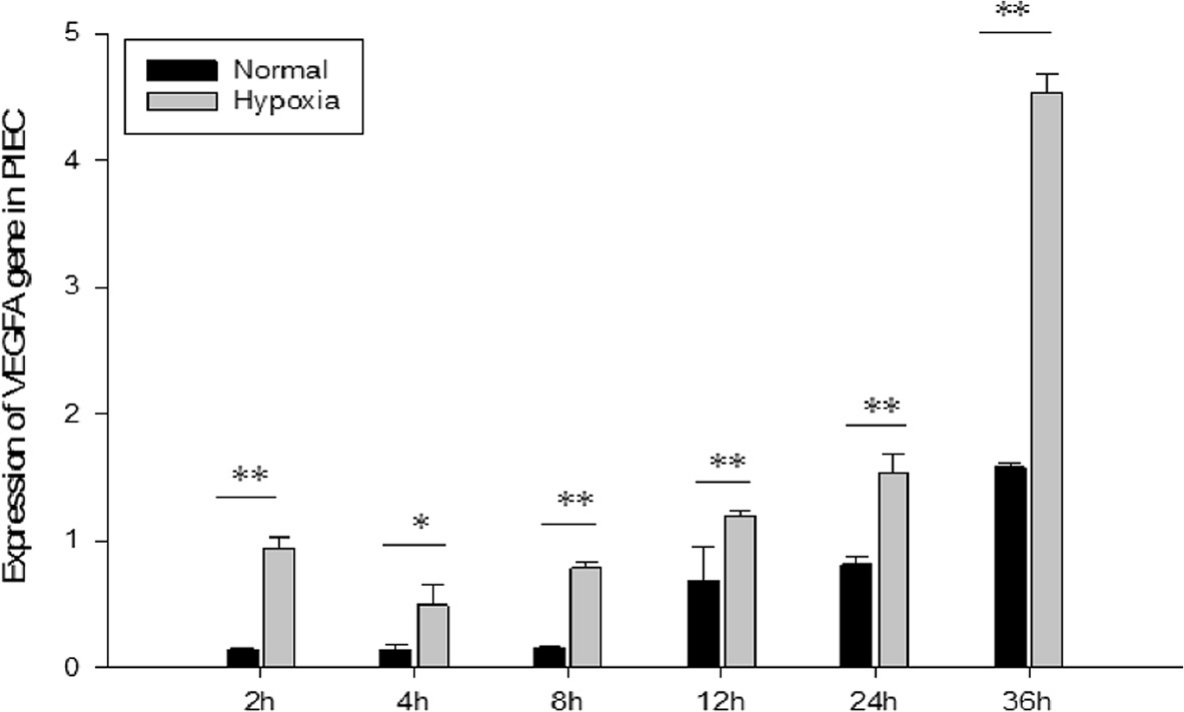
Quantitative expression of *VEGFA* mRNA in endothelial cells. Each bar represents mean ± S.E. * Significant difference (*P* < 0.05), ** Extreme significant difference (*P* < 0.01) (*n* = 3)

Expression of *VEGFA* mRNA in endothelial cells is shown in Fig. 5. At all time points, expression of *VEGFA* mRNA in vitro was higher under hypoxic condition than under normoxic condition (*P <* 0.05). Under both normoxic and hypoxic conditions, expression of *VEGFA* mRNA had an increased trend after 4 h over time.

## Discussion

*VEGFA* is a pivotal angiogenic factor that binds to specialized receptors on the surface of endothelial cells and induces them to generate new vessels [12]. *VEGFA* expression was modulated by *HIF-1* through binding to promoters of hypoxia response elements (HREs) [13, 14].

We found 5 SNPs in the *VEGFA* gene. TP, as well as the other pig breeds, exhibited relatively large polymorphisms at the 5 loci, although the distinction between frequency distributions was not significant. No SNPs were detected in the coding region of the *VEGFA* gene. The mRNA sequence of *VEGFA* was highly conserved among pig breeds, which is consistent with previous studies showing that both the mRNA sequence and protein domain of human *VEGFA* gene were conserved [15]. Thus, the biological function of *VEGFA* is primarily regulated by controlling its expression. The results also indicated that there might be other regulatory mechanisms (for example of epigenetic regulation) in the region or functional SNPs in long-distance regions. It was a pending work what SNPs or what other regulatory mechanisms could regulate the gene expression and have roles on hypoxic adaptation in Tibetan pig.

The heart plays an important role in adaptation to hypoxia. It has been reported that decreased cardiac *VEGFA* signaling interferes with myocardial angiogenesis. This results in local ischemia, which triggers cardiomyocyte damage and heart failure [16, 17]. In the present study, *VEGFA* expression in heart tissue was significantly higher in TP compared with Yorkshire under hypoxia at high altitudes. To adapt to a hypoxic environment, TP increased expression of the *VEGFA* gene in vivo and changed their cardiovascular response to hypoxia. The increased *VEGFA* expression might increase blood flow and enhance cardiac pumping [18, 19].

In the early phase of liver regeneration, proliferating hepatocytes showed hypoxia-induced *VEGFA* expression, which initiates proper blood flow through the liver [20]. Our results consistently showed that expressions of *VEGFA* mRNA and protein in liver were significantly higher in TP than in YH and YL, which indicated that the TP might improve blood flow in liver tissue to adapt to hypoxia.

*VEGFA* plays a crucial role in the kidney, where it is produced primarily by glomerular epithelial cells (podocytes) and is also found in epithelial cells [21, 22]. In mice, specific overexpression or deletion of the *VEGFA* gene in podocytes results in glomerular dysfunction [23, 24]. Moreover, *VEGFA* acts as an autocrine growth factor on both proliferating and differentiating glomerular visceral epithelial cells (podocytes) [24] and has roles in prolonged survival and resistance to apoptosis [25]. In the present study, TP showed a high expression level of the *VEGFA* gene, suggesting that *VEGFA* plays a pivotal role in the maintenance of glomerular integrity under hypoxia in the kidneys of pigs.

## Conclusion

We found that the mRNA sequence of the pig *VEGFA* gene was conserved among pig breeds, which indicated the biological function of the gene was primarily regulated by differential expression. Only five SNPs (G-2745C, G-2442A, G-2435deletion, C-1773 T and T-1010C) were found in the 5′-flanking region of length of 2693 bp upstream from the initiation codon of the *VEGFA* gene among the TP, YL, and DN populations. However, further studies are required to identify the site that can regulate the gene expression in pig. The Tibetan pig had considerably high expressions of the *VEGFA* gene in heart and liver tissues in high-altitude environment. The increased *VEGFA* expression might be one way of genetic adaptation to hypoxia in high-altitude, through promoting endothelial cells proliferation, angiogenesis and maintaining vascular permeability. Further research on molecular mechanisms of the *VEGFA* for hypoxic adaptation was a pending work in Tibetan pig.

## Abbreviations

DN: Diannan small-ear pig
*eNOS*: endothelial nitric oxide synthase
*HIF-1α*: hypoxia-inducible factor 1α
HREs: hypoxia response elements
PIEC: pig iliac endothelial cells
qRT-PCR: quantitative real-time PCR
SDS-PAGE: sodium dodecyl sulfate polyacrylamide gel electrophoresis
SNPs: single nucleotide polymorphisms
TP: Tibetan pig
*VEGFA*: vascular endothelial growth factor A
*VEGFR-1*: *VEGF* receptor 1
*VEGFR-2*: *VEGF* receptor 2
YH: Yorkshire pigs that migrated to high altitudes
YL: Yorkshire pigs that lived at low altitudes
